# Fucoxanthin inhibits the proliferation of MOLM13 cells by targeting AKT to disrupt glucose metabolism

**DOI:** 10.3389/fphar.2025.1601281

**Published:** 2025-07-15

**Authors:** Tingting Niu, Ying Chen, Mengmeng Sun, Cong Shi, Duobing Zou, Wei Wu, Yuzhan Chen, Juanjuan Chen, Haimin Chen, Guifang Ouyang, Qitian Mu

**Affiliations:** ^1^ Laboratory of Stem Cell Transplantation, The First Affiliated Hospital of Ningbo University, Ningbo, Zhejiang, China; ^2^ Collaborative Innovation Center for Zhejiang Marine High-efficiency and Healthy Aquaculture, Ningbo University, Ningbo, Zhejiang, China; ^3^ Department of Hematology, The First Affiliated Hospital of Ningbo University, Ningbo, Zhejiang, China

**Keywords:** fucoxanthin, acute myeloid leukemia, Akt, GLUT1, glucose metabolism

## Abstract

Fucoxanthin is a natural carotenoid that has remarkable anti-tumor effects and an excellent safety profile. Here, we combined molecular docking, dynamics simulations, and functional assays (CCK-8, flow cytometry, glucose/ATP detection) to decipher the mechanism of Fucoxanthin on FLT3-ITD AML cells. Fucoxanthin (25 μM) reduced MOLM13 (FLT3-ITD) cell viability by 63.6% (*P* < 0.01), inducing G0/G1 arrest via CDK4 downregulation and apoptosis through Bcl2 suppression. Fucoxanthin also inhibited the glucose uptake, GLUT1 membrane translocation, and ATP production. Mechanistically, fucoxanthin directly bound to AKT and inhibited its kinase activity by 57.9%, while AKT overexpression rescued the glucose/ATP suppression (*P* < 0.05). Molecular dynamics revealed critical interactions between fucoxanthin and Phe-236/Lys-179. These results suggest that fucoxanthin may selectively target AKT-dependent glucose metabolism in MOLM13 cells, warranting further investigation into its role in addressing metabolic alterations in FLT3-ITD AML.

## 1 Introduction

Acute myeloid leukemia (AML) is the most common adult leukemia and has a poor prognosis, with a 5-year overall survival (OS) rate of 24%. FMS-like tyrosine kinase 3 (FLT3)-Internal Tandem Duplication (ITD) mutations occur in 25%–30% of AML cases, and they are associated with a particularly poor prognosis (Anabtawi et al., 2025). In these cases, constitutive FLT3 activation drives leukemogenesis primarily through the phosphoinositide 3-kinase (PI3K)/protein kinase B (AKT) and signal transducer and activator of transcription5 (STAT5) pathways (Arwanih et al., 2024). As AKT is a key node in FLT3 downstream signaling, its hyperactivation enhances the phosphorylation of mechanistic target of rapamycin (mTOR), Bcl2-associated agonist of cell death (BAD), and other effectors, which then promote leukemic cell survival, proliferation, and metabolic reprogramming. These events lead to hyperleukocytosis, early relapse, and FLT3-ITD cases have significantly poorer chemotherapy response than FLT3-WT cases (Chen et al., 2023; Gu et al., 2024). Current FLT3 inhibitors (midostaurin, gilteritinib) provide only modest survival benefits (median OS 3–4 months) due to resistance and toxicity (myelosuppression, cardiotoxicity) (Araújo et al., 2025; Ge et al., 2023). Given these limitations, targeting AKT or its associated pathways has emerged as a promising alternative strategy, prompting exploration of novel approaches such as covalent FLT3 inhibitors (e.g., FF-10101), metabolic targeting of the AKT-glucose transporter proteins 1 (GLUT1) axis, and dual FLT3/BCL-2 inhibition (Ge et al., 2022). Notably, natural compounds capable of modulating AKT signaling, such as the carotenoid fucoxanthin, may offer a complementary therapeutic avenue.

Natural products have emerged as cancer treatment drugs since the 1950s (Kashyap et al., 2021; Newman and Cragg, 2020). Carotenoids are organic pigments found in many foods, and there are over 40 varieties of carotenoids in the human diet. Carotenoids have various health benefits and have been used in treating various types of cancer, including leukemia (Zhang et al., 2022), colorectal cancer (Terasaki et al., 2023), and gastric cancer (Wang et al., 2023). Fucoxanthin is a naturally occurring carotenoid derived from seaweed, and it is predominantly found in the chloroplasts of macroalgae and microalgae (Sathishkumar and Sathuvan, 2025; Takaichi, 2025). The molecular structure of fucoxanthin sets it apart from other carotenoids, as it has a unique 5, 6-monoepoxide ring and an allene (Liu et al., 2020; Yuan et al., 2023). These unique structural attributes endow fucoxanthin with enhanced bioactivity compared to conventional carotenoids as well as the potential for selectively targeting oncogenic signaling pathways (Koch et al., 2024; Lau and Kwan, 2022).

Fucoxanthin has demonstrated broad anti-tumor activity by targeting the PI3K/AKT pathway, which is a key vulnerability in FLT3-ITD-driven AML. In treating hematological tumors, fucoxanthin can independently induce apoptosis in adult T-cell leukemia and HL-60 cells, and it can enhance the cytotoxicity of imatinib and doxorubicin when used in combination (Almeida et al., 2018; Ishikawa et al., 2008). Critically, its ability to inhibit AKT signaling (Sathishkumar and Sathuvan, 2025) aligns with the therapeutic needs of FLT3-mutated AML, where constitutive PI3K/AKT activation promotes chemoresistance (Kurosu et al., 2013). Fucoxanthin also suppresses PI3K/AKT/mTOR in other cancers (e.g., glioblastoma (Sathishkumar and Sathuvan, 2025), Human Pharyngeal Squamous Cell Carcinoma (Du et al., 2024)), though its specific effects on FLT3-ITD clones require further validation. Notably, in toxicological and metabolic studies, no adverse effects associated with the administration of fucoxanthin have been identified (Beppu et al., 2009; Lau and Kwan, 2022; Méresse et al., 2020; Mohibbullah et al., 2022; Zhang et al., 2015). In view of its promising anti-tumor effects and safety profile, fucoxanthin represents a candidate worthy of further exploration for FLT3-mutated subtypes.

The AKT signaling pathway has a critical role in regulating glucose metabolism. AKT mediates the translocation of GLUT1 to the cell membrane and maintains glucose homeostasis (Feng et al., 2020). It also promotes the translation of GLUT1 via mTOR complex 1 (mTORC1), which alters the GLUT1 expression (Siska et al., 2016). It stimulates other glycolytic enzymes either through direct phosphorylation or by modulating the function of hexokinase, phosphofructokinase-1, and phosphofructokinase-2 (Herschbein and Liesveld, 2018). Recent research has demonstrated a strong correlation between disrupted glucose metabolism and the development of AML (Zhao et al., 2024). The stronger glycolysis activated by higher glucose uptake improves the survival of cancer cells (Deng et al., 2023; Ye et al., 2018). Taken together, targeting glucose metabolism via AKT may be used to develop new AML therapies.

This study elucidates the antileukemic mechanism of fucoxanthin in FLT3-ITD AML cells through an integrated computational and experimental approach. Building upon the current demand for safer targeted therapies and emerging evidence of metabolic vulnerabilities in AML, we i) identified the critical binding residues (Phe-236/Lys-179) of fucoxanthin-AKT interaction, and ii) demonstrated the dual mechanism of fucoxanthin, which involves CDK4-mediated cell cycle arrest and GLUT1/ATP metabolic reprogramming. We propose that fucoxanthin’s FLT3-independent targeting of AKT-driven metabolic vulnerabilities may represent a novel strategy to circumvent the intrinsic resistance pathways in FLT3-ITD AML. These findings provide a novel strategy for developing precision therapies targeting both proliferative and metabolic pathways in high-risk AML.

## 2 Materials and methods

### 2.1 Materials

Human acute myeloid leukemia cells MOLM13, OCI-AML2, NB4, HL-60, and U937 were kindly provided by Professor Jie Jin from the Institute of Hematology at the First Affiliated Hospital of Zhejiang University. KG-1α cells were obtained from the American Type Culture Collection. MOLM13, OCI-AML2, and KG-1α cells were maintained in Iscove’s Modified Dulbecco Medium (IMDM) supplemented with 10% fetal bovine serum (FBS). U937, NB4, and HL-60 cells were incubated in PRMI 1640 medium. All cells were cultured at 37°C in a humidified atmosphere containing 5% CO_2_.

In addition, mononuclear cells were isolated from the bone marrow samples collected from patients diagnosed with AML by Ficoll–Hypaque (Sigma-Aldrich, United States) density gradient centrifugation. Anticoagulated whole blood or marrow was mixed with an equal volume of PBS containing 2% FBS, carefully layered over Ficoll solution, and centrifuged at 400 × g for 30 min. The buffy coat layer was then collected, washed twice with PBS (300 × g, 10 min each), and finally resuspended in RPMI-1640 medium supplemented with 10% FBS. Cell viability (>95%) was confirmed by trypan blue exclusion. Written informed consent was obtained from all participating patients. The study protocol was approved by the Ethics Committee of the First Affiliated Hospital of Ningbo University, China (NBU-2023-081).

Algae-derived fucoxanthin (99.47% purity) was purchased from MedChem Express (Monmouth Junction, NJ, United States).

### 2.2 Cell viability

Fucoxanthin (1.56–200 μM; 2-fold dilutions) was tested on MOLM13 cells (5 × 10^4^/well) for 24 h. The IC_50_ value was calculated using a four-parameter logistic model, and 25 μM (1.5× IC_50_) was selected as the optimal dose based on preliminary kinetics data. MOLM13, NB4, HL-60, OCI-AML2, KG-1α, and U937 cells were seeded in 96-well plates at a density of 1 × 10^4^ cells per well. The cells were then treated with 6.25, 12.5 and 25 μM natural carotenoids (fucoxanthin, astaxanthin, and lycopene) for 24 h. The mononuclear cells isolated from bone marrow were treated with 25 μM fucoxanthin for 24 or 48 h. Subsequently, the cell counting kit-8 solution (10 μL) (NCM Biotech, Shanghai, China) was added to each well and the cells were incubated for an additional 2 h, and the spectrophotometric absorbance at 450 nm was measured. All experiments were performed in triplicate. The results were expressed as a percentage of the control (Zhang et al., 2025).

### 2.3 Apoptosis

Apoptosis induction was assessed using an annexin V-APC/7-AAD apoptosis kit (Liankebio, Hangzhou, China). After treatment with fucoxanthin (6.25, 12.5 and 25 μM) for 24 h, cells were washed twice with cold phosphate-buffered saline (PBS), suspended in the binding buffer, incubated with annexin V-APC and 7-AAD for 15 min, then analyzed by flow cytometry using a FACScan flow cytometer (Becton Dickinson, San Diego, CA, United States) (Wu et al., 2022).

### 2.4 Cell cycle arrest

Cell cycle arrest analysis was performed using a propidium iodide DNA staining kit (Liankebio, Hangzhou, China). After treatment with fucoxanthin (6.25, 12.5 and 25 μM) for 24 h, cells were harvested, fixed with 70% ethanol (1 mL per 1 × 10^6^ cells) at −20°C overnight, and hydrated with PBS at room temperature for 15 min. The DNA staining solution (1 mL) was then added, and the cells were incubated at room temperature in the dark for 30 min before analysis by flow cytometry using a FACScan flow cytometer (Becton Dickinson, San Diego, CA, United States) (Li et al., 2022).

### 2.5 Glucose uptake

The fluorescent d-glucose analog 2-(N-(7-nitrobenz-2-oxa-1,3-diazol-4-yl)amino)-2-deoxyglucose (2-NBDG) (MedChemExpress, Monmouth Junction, NJ, United States) was employed as a fluorescent indicator to assess glucose uptake. MOLM13 cells were treated with fucoxanthin (6.25, 12.5 and 25 μM) for 24 h, harvested and washed with PBS, starved in glucose-free medium for 1 h, then incubated with 2-NBDG (100 μM) at 37°C under 5% CO_2_ for 30 min. The cells were then harvested by centrifugation, washed with cold PBS twice, and the 2-NBDG signal was analyzed using a FACScan flow cytometer (Becton Dickinson, San Diego, CA, United States) (Qiu et al., 2025).

### 2.6 ATP content

MOLM13 cells were seeded in 6-well plates at a density of 1 × 10^6^ cells per mL, treated with fucoxanthin (6.25, 12.5 and 25 μM) for 24 h, and washed twice with PBS. The ATP content was measured using an ATP assay kit (Beyotime, Shanghai, China) following the manufacturer’s instructions. Specifically, cells were lysed with ice-cold lysis buffer (200 μL per 1 × 10^6^ cells, 6-well plate equivalent), vortexed for 10 s, incubated on ice for 10 min, and centrifuged at 12,000 × g and 4°C for 5 min; the supernatants were collected for analysis. ATP standards (0.01–10 μM) were prepared by serial dilution in lysis buffer, and the working solution was created by mixing the ATP detection reagent with the assay diluent (1:9 v/v). For detection, the working solution (100 μL) was added to luminometer plates, incubated at room temperature for 5 min to permit background ATP hydrolysis, followed by the injection of the sample or standard (20 μL). Luminescence (RLU) was then measured within 2 s (Wang et al., 2022).

### 2.7 AKT kinase activity assay

AKT kinase activity was measured using a commercial colorimetric assay kit (Genmed Scientifics Inc., United States) with minor modifications. Cells cultured in 6-well plates were lysed in ice-cold lysis buffer (100 μL), vortexed for 15 s, and incubated on ice for 30 min. The lysates were centrifuged (12,000 × g, 4°C, 10 min), and the supernatants were collected. The total protein concentration was determined via BCA assay (Easy II Quantitative Kit, TransGen Biotech, Beijing, China). For the reaction, the total protein (50 μg) was combined with the assay buffer to attain a final volume of 200 μL, and the mixture was incubated at 30°C for 40 min. The enzyme activity was determined by monitoring NADH oxidation at 340 nm, and one unit of AKT activity was defined as the oxidation of 1 μmol NADH/min/mg protein at pH 7.5°C and 30°C (Fang et al., 2021).

### 2.8 Molecular docking

The crystal structure of the AKT protein (PDB:4GV1) was downloaded from the PDB database (http://www.rcsb.org/). The 3D structure of fucoxanthin was obtained from the PUBCHEM database (https://pubchem.ncbi.nlm.nih.gov/), and energy minimization was performed using the MMFF94 force field. The AutoDock Vina 1.1.2 software was used for molecular docking. The receptor protein was processed using PyMol 2.5 to remove water molecules, salt particles, and small molecules. The center of the box (25 × 25 × 25 Å3) was set to the centroid of the ligand in the original crystal structure. All processed small molecules and the receptor protein were, using ADFRsuite 1.03, converted into the PDBQT format required for docking. During docking, the exhaustiveness of the global search was set to 32, and the default settings were used for other parameters. The docked conformation with the highest score was considered the binding conformation, and the docking results were visualized using PyMol 2.5 (Wu et al., 2021).

### 2.9 Molecular dynamics

Molecular dynamics simulations were performed using the AMBER 18 software. The system first underwent energy minimization using a combination of 2,500 steps of steepest descent and 2,500 steps of conjugate gradient method, and it was then heated at a constant rate over 200 ps from an initial temperature of 0 K to the desired simulation temperature of 298.15 K while the volume remained fixed. Once the system reached 298.15 K, a 500 ps simulation was performed in the NVT ensemble to allow the solvent molecules to distribute evenly within the solvent box. Afterwards, a 500 ps equilibrium simulation of the entire system was performed under NPT (constant temperature and pressure) conditions.

Two complex systems were then simulated under periodic boundary conditions for a total of 30 ns each using the NPT ensemble. During these simulations, a non-bonded cutoff distance of 10 Å was applied to determine the interactions between atoms. The particle mesh Ewald (PME) method was employed to calculate long-range electrostatic interactions. The SHAKE algorithm was utilized to constrain the length of hydrogen bonds in the system. To control the temperature, Langevin dynamics was applied with a collision frequency of 2 ps^−1^. The system pressure was maintained at 1 atm, and the integration time step was 2 fs. The trajectories of the simulations were saved every 10 ps. The binding free energy between the protein and the ligand was calculated using the MM/GBSA method as follows:

$$∆G_{bind}=∆G_{complex}-\left(∆G_{receptor}+∆G_{ligand}\right)$$

$$=∆E_{\mathrm{int}ernal}+∆E_{VDW}+∆E_{elec}+∆G_{GB}+∆G_{SA}$$

where Δ*G* is the Gibbs free energy, and Δ*E*_internal_, Δ*E*_VDW_, and Δ*E*_elec_ represent the internal energy, van der Waals interactions, and electrostatic interactions, respectively. The internal energy involves the bond energy (*E*_bond_), the angle energy (*E*_angle_), and the torsional energy (*E*_torsion_). The solvation-free energy is collectively referred to as Δ*G*_*GB*_ and Δ*G*_*SA*_, where *G*_*GB*_ represents the polar solvation free energy and *G*_*SA*_ represents the non-polar solvation free energy. The generalized Born (GB) model developed by Nguyen et al. (*igb* = 8) was used to calculate Δ*G*_GB_. The non-polar solvation free energy (Δ*G*_SA_) was calculated as the product of the surface tension and the solvent-accessible surface area (SASA), i.e., Δ*G*_SA_ = 0.0072 × ΔSASA (Needham et al., 2016; Salomon-Ferrer et al., 2013).

### 2.10 Cell model with AKT overexpression

The AKT overexpression plasmid pEX-3(pGCMV/MCS/Neo)-AKT and the empty vector plasmid pEX-3(pGCMV/MCS/Neo) were purchased from GenePharma (Shanghai, China) and transfected into HEK-293T cells using Lipofectamine^®^ 3,000 (Thermo Fisher Scientific, Waltham, MA, United States) (Ma et al., 2014). Twenty-four hours after the transfection, the cells were treated with fucoxanthin and incubated for an additional 24 h. The cells were then collected, and the expression levels of poly ADP ribose Polymerase (PARP) and GLUT1 were measured by Western blot. In addition, the AKT kinase activity and the ATP levels were quantified using the corresponding assay kits.

### 2.11 Western blot

The total protein was extracted using a total protein extraction kit (Beyotime, Shanghai, China) following the manufacturer’s instructions. The protein concentration was determined using the BCA Kit (TransGen, Beijing, China). To begin the Western blot analysis, equal amounts of proteins (30 μg) from the whole cell lysates were separated using 10% sodium dodecyl sulfate-polyacrylamide gel (SDS-PAGE) and transferred to polyvinylidene fluoride (PVDF) membranes. The membranes were blocked with 5% skim milk for 2 h at room temperature, incubated at 4°C with the primary antibodies overnight, then incubated with the appropriate secondary antibodies at room temperature for 1 h. The immunoreactive bands were visualized by electrochemiluminescence using NcmECL Ultra-HRP Substrate (New Cell & Molecular Biotech Co., Ltd., Suzhou, China). The band intensity was quantified using ImageJ with β-actin as the control. The primary antibodies included Bcl-2 (1:1000), Bax (1:1000), GLUT1 (1:1000), PARP (1:1000), CDK4 (1:1000), CDK6 (1:1000), and β-actin (1:2000) (Cell Signaling Technology, Danvers, MA, United States). The secondary antibodies included horseradish peroxidase (HRP)-conjugated mouse anti-rabbit IgG (1:5000) and goat anti-mouse IgG (1:5000) antibodies (Cell Signaling Technology, Danvers, MA, United States) (Niu et al., 2018).

### 2.12 Statistical analysis

Data were presented as the mean ± standard deviation (SD). Statistical analyses were conducted using SPSS 16.0 (SPSS Inc., Chicago, IL, United States). The statistical significance was assessed using one-way ANOVA with the Tukey multiple comparison test. Differences were considered statistically significant when *P* < 0.05.

## 3 Results

### 3.1 Fucoxanthin inhibited the growth of AML cells

**FIGURE 1:**
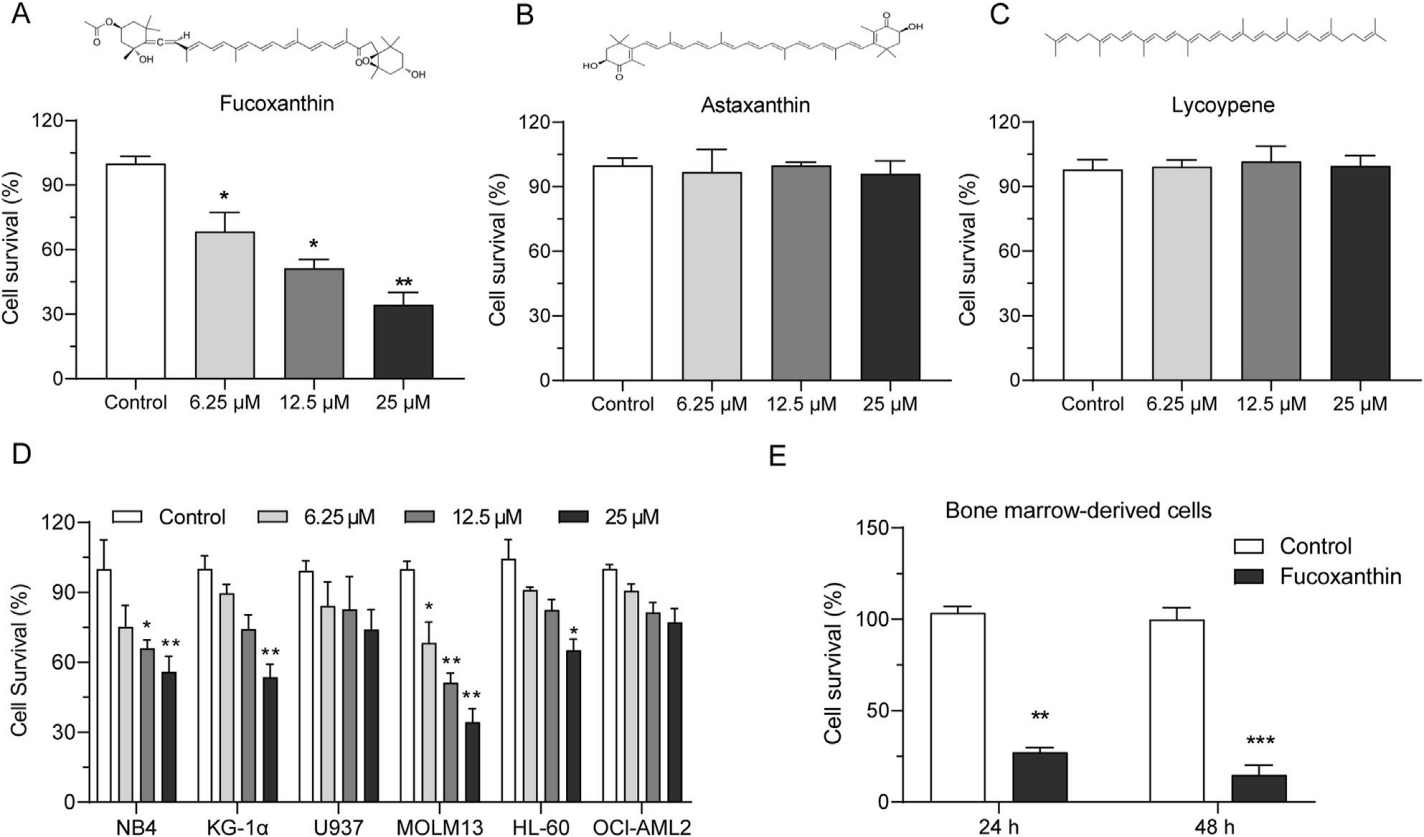
Fucoxanthin inhibited the growth of AML cells. Cell viability after incubation of MOLM13 cells with **(A)** fucoxanthin, **(B)** astaxanthin, and **(C)** lycopene at 6.25, 12.5, and 25 μM for 24 h. **(D)** Incubation of different AML cell lines with fucoxanthin (6.25, 12.5, and 25 μM for 24 h). **(E)** Incubation of bone marrow-derived primary cells with 25 μM fucoxanthin for 24 and 48 h. Data are presented as the mean ± SD of three experiments. ^*^ *P* < 0.05, ^**^ *P* < 0.01, ^***^ *P* < 0.001 (n = 3) compared to the control.

Among the three tested natural carotenoids, astaxanthin and lycopene did not have a noticeable effect on MOLM13 cells, whereas fucoxanthin significantly inhibited the growth of MOLM13 cells (Figures 1A–C). The survival of MOLM13 cells reduced by 63.6% when they were treated with 25 μM fucoxanthin. Figure 1D shows that among the AML cell lines in the growth test, MOLM13 cells were inhibited by fucoxanthin most effectively, followed by KG-1α and NB4 cells, while no apparent effect was detected for OCI-AML2 or U937 cells (*P* > 0.05). In addition, when bone marrow-derived primary cells were treated with 25 μM fucoxanthin, the cell survival decreased by 72.9% after 24 h and by 85.2% after 48 h (Figure 1E).

### 3.2 Fucoxanthin promoted cell cycle arrest and apoptosis in MOLM13 cells

**FIGURE 2:**
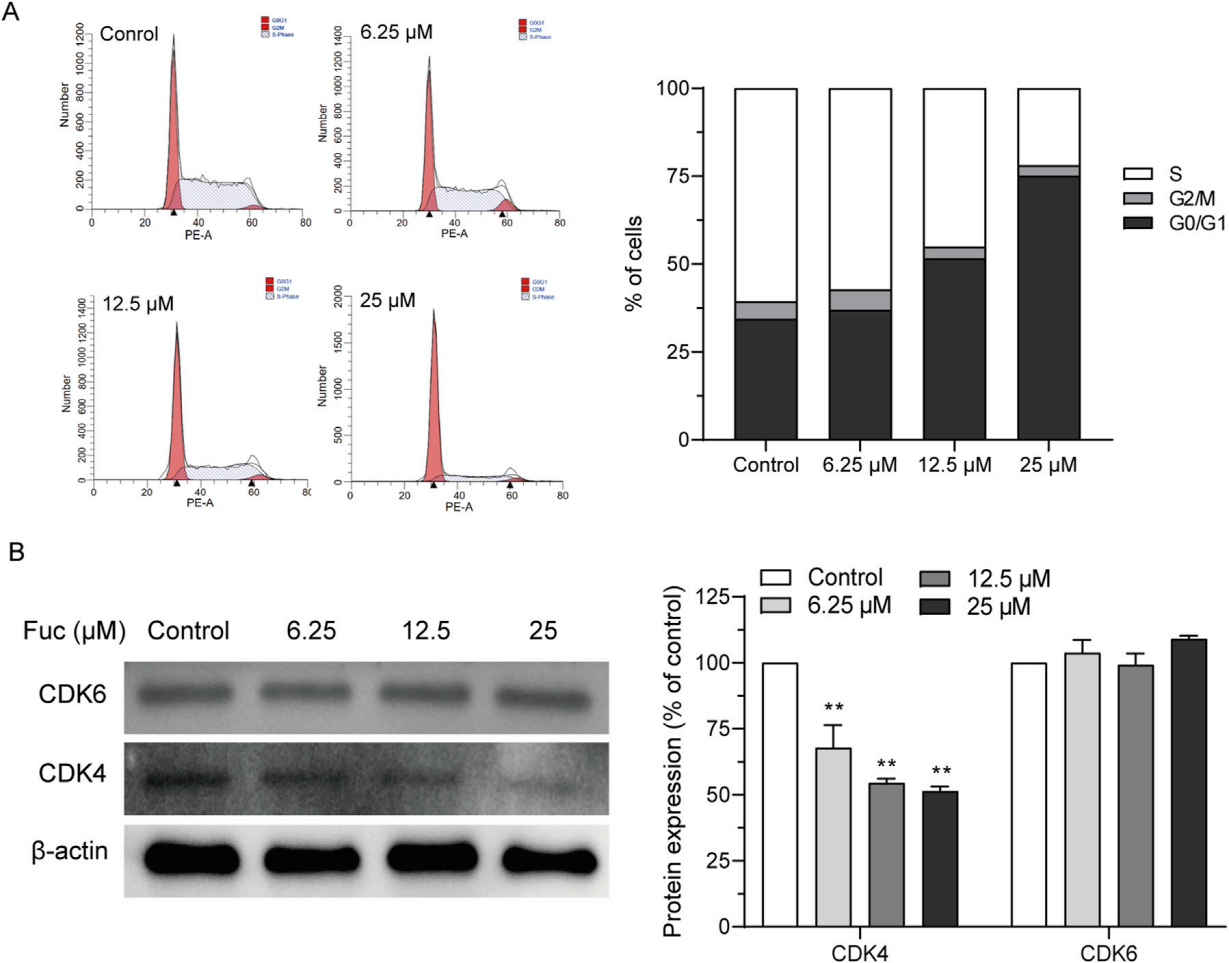
Fucoxanthin promoted cell cycle arrest in MOLM13 cells. **(A)** Effect of fucoxanthin on cell cycle arrest in MOLM13 cells. **(B)** Effect of fucoxanthin on CDK4 and CDK6 expression. Data are presented as the mean ± SD of three experiments. ^**^ *P* < 0.01 (n = 3) compared to the control.

After MOLM13 cells were treated with 25 μM fucoxanthin for 24 h, compared to the control group, the proportion of cells in the G0/G1 phase increased by 118.72%, whereas the proportion of cells in the S phase decreased by 63.8% (Figure 2A). The expression levels of CDK4 and CDK6 proteins, which are involved in cell cycle regulation, were investigated to examine the mechanism underlying the observed cell cycle arrest. Interestingly, while the treatment with 25 μM fucoxanthin for 24 h decreased the CDK4 expression in MOLM13 cells by 48.6%, the CDK6 expression was not affected (Figure 2B). Thus, it could be inferred that fucoxanthin induced cell cycle arrest in MOLM13 cells at the G0/G1 phase by inhibiting the expression of the CDK4 protein.

**FIGURE 3:**
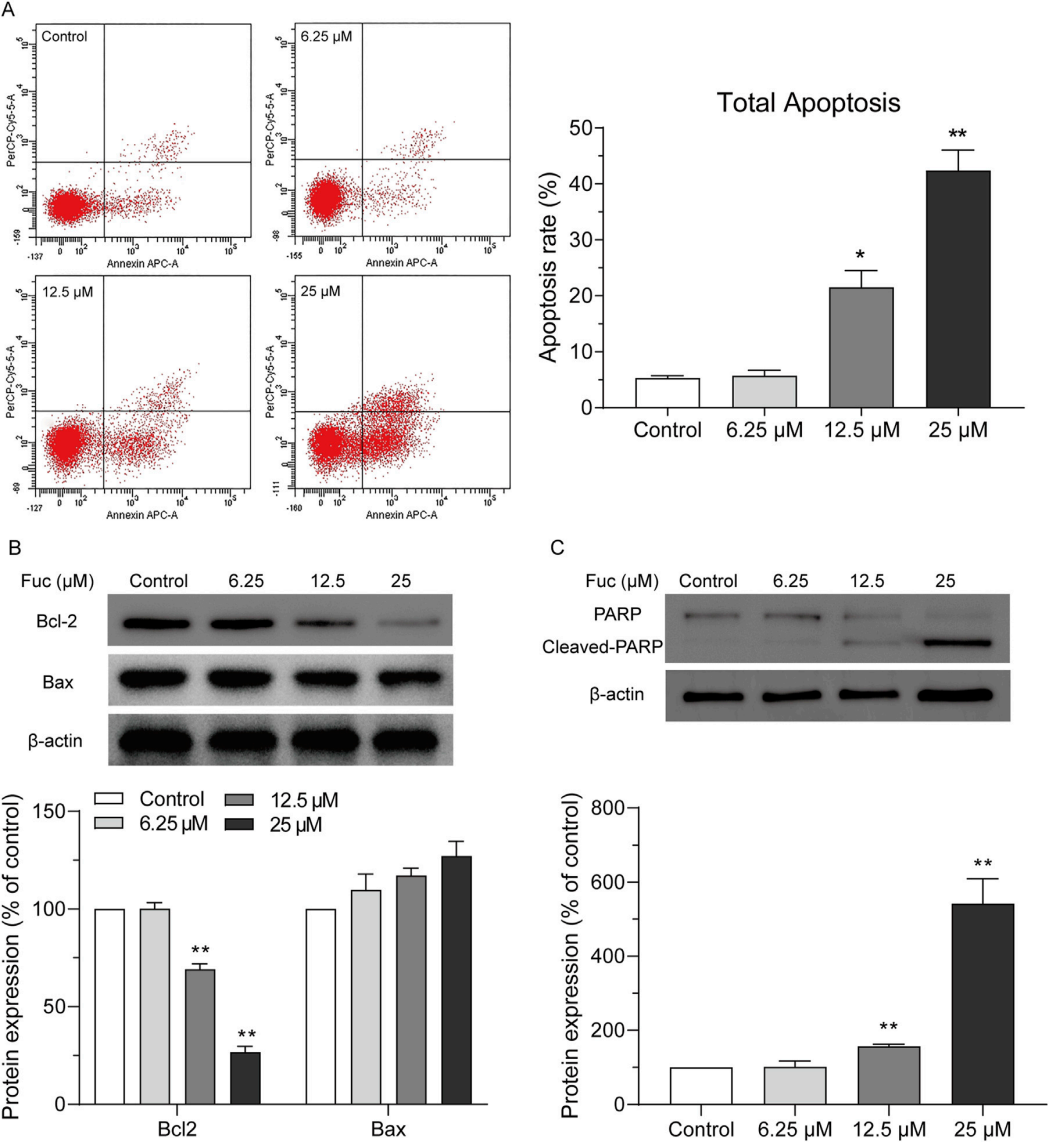
Fucoxanthin promoted apoptosis in MOLM13 cells. **(A)** Effect of fucoxanthin on the apoptosis of MOLM13 cells. **(B)** Effect of fucoxanthin on the expression of Bcl2 and Bax proteins. **(C)** Effect of fucoxanthin on PARP cleavage. Data are presented as the mean ± SD of three experiments. ^*^ *P* < 0.05, ^**^ *P* < 0.01 (n = 3) compared to the control.

Cells may undergo apoptosis to trigger programmed death after prolonged cell cycle arrest. Indeed, the apoptosis of MOLM13 cells increased with rising concentration of fucoxanthin used in the incubation (Figure 3A). Interestingly, the treatment with 25 μM fucoxanthin, which raised the apoptosis rate of MOLM13 cells up to 38.3%, decreased the Bcl2 expression by 75.3% (*P* < 0.01) but did not affect the Bax expression (Figure 3B), and it increased the cleavage of the PARP protein remarkably (Figure 3C, *P* < 0.01). Therefore, fucoxanthin induced apoptosis in MOLM13 cells by enhancing the cleavage of the PARP protein and inhibiting the expression of the Bcl2 protein.

### 3.3 Fucoxanthin directly targeted AKT to inhibit its kinase activity

**FIGURE 4:**
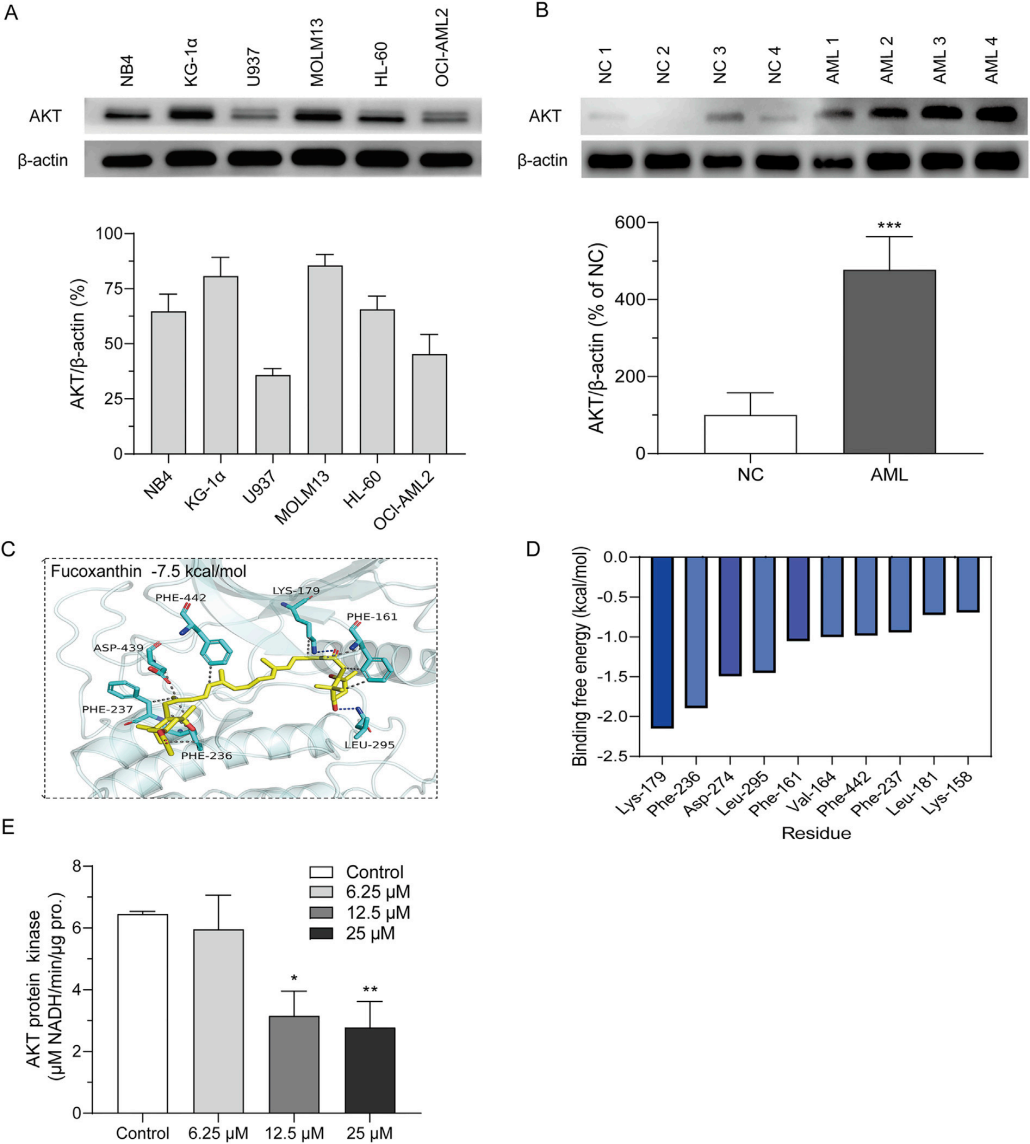
Fucoxanthin directly targeted AKT to inhibit its kinase activity. **(A)** AKT expression levels of different cell lines. **(B)** AKT expression levels of peripheral blood cells (NC) and primary AML cells derived from bone marrow (AML). **(C)** Molecular docking of fucoxanthin and the AKT protein. **(D)** Molecular dynamics analysis of the key amino acids in the binding of fucoxanthin to AKT. **(E)** The activity of AKT kinase in MOLM13 cells. Data are presented as the mean ± SD of three experiments. ^*^ *P* < 0.05, ^**^ *P* < 0.01, ^***^ *P* < 0.001 (n = 3) compared to the control.

MOLM13 cells had higher levels of the AKT protein than other cell lines, and U937 and OCI-AML2 cells had the lowest levels of the AKT protein (Figure 4A). In addition, the AKT expression was significantly stronger in the bone marrow-derived primary cells than in the peripheral blood cells (Figure 4B). Molecular docking was used to assess the binding between fucoxanthin and AKT. Figure 4C illustrates that fucoxanthin is capable of specific binding with AKT, and the affinity is −7.5 kcal/mol. The active pocket on the AKT protein that envelops fucoxanthin features Phe-237, Phe-236, Asp-439, Phe-442, Phe-161, Lys-179, and Leu-295. Fucoxanthin engages Phe-237, Phe-236, Asp-439, Phe-442, and Phe-161 through hydrophobic interactions and Lys-179 and Leu-295 through hydrogen bonding. Molecular dynamics simulation shows that fucoxanthin has a binding energy of −30.38 kcal/mol to the AKT protein. The amino acids Lys-179 and Phe-236 are primarily involved in the binding, and they contribute −2.15 and −1.89 kcal/mol to the binding energy, respectively (Figure 4D). Fucoxanthin decreased the kinase activity of AKT in a concentration-dependent manner (Figure 4E), and the AKT kinase activity dropped by 57.9% (*P* < 0.01) after MOLM13 cells were treated with 25 μM fucoxanthin for 24 h.

### 3.4 Fucoxanthin inhibited cellular ATP production in MOLM13 cells

**FIGURE 5:**
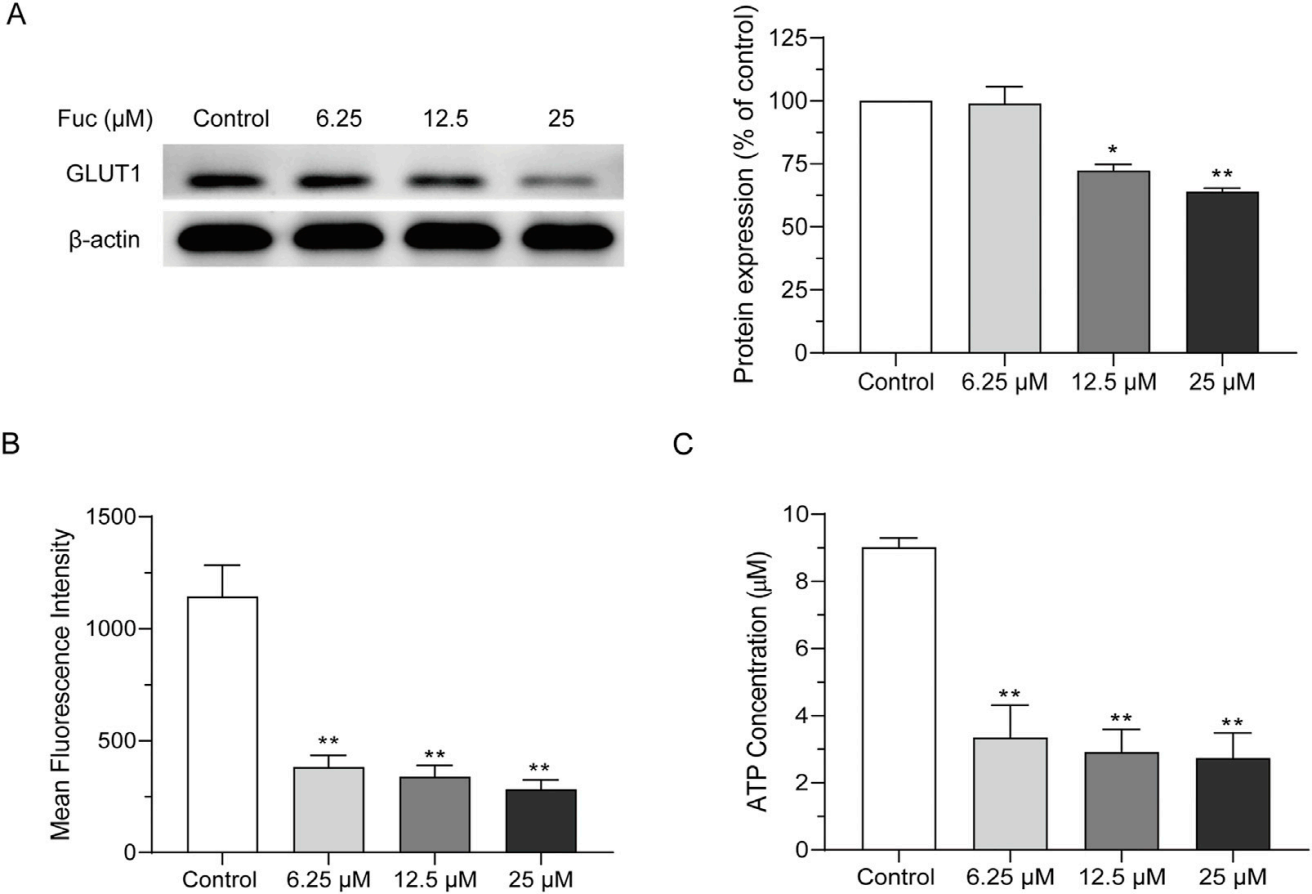
Fucoxanthin inhibited cellular ATP production in MOLM13 cells. **(A)** Effect of fucoxanthin on GLUT1 expression. **(B)** Effect of fucoxanthin on glucose absorption in MOLM13 cells. **(C)** Effect of fucoxanthin on ATP production in MOLM13 cells. Data are presented as the mean ± SD of three experiments. ^*^ *P* < 0.05, ^**^ *P* < 0.01 (n = 3) compared to the control.

The GLUT1 expression of MOLM13 cells dropped significantly after incubation with fucoxanthin at a higher concentration (Figure 5A), and their glucose absorption also declined with rising fucoxanthin concentration in a dose-dependent manner (Figure 5B). In addition, fucoxanthin also significantly reduced the cellular ATP content (Figure 5C). After MOLM13 cells were incubated with 25 μM fucoxanthin for 24 h, the GLUT1 expression decreased by 36.1% (*P* < 0.01), the glucose absorption dropped by 75.35% (*P* < 0.01), and the intracellular ATP fell by 71.7% (*P* < 0.01). Presumably, these results suggested that fucoxanthin may impair glucose metabolism in MOLM13 cells, possibly through inhibition of GLUT1-mediated glucose transport, as evidenced by reduced glucose uptake and subsequent ATP depletion.

### 3.5 Fucoxanthin targeted AKT to inhibit ATP production and PARP activation

**FIGURE 6:**
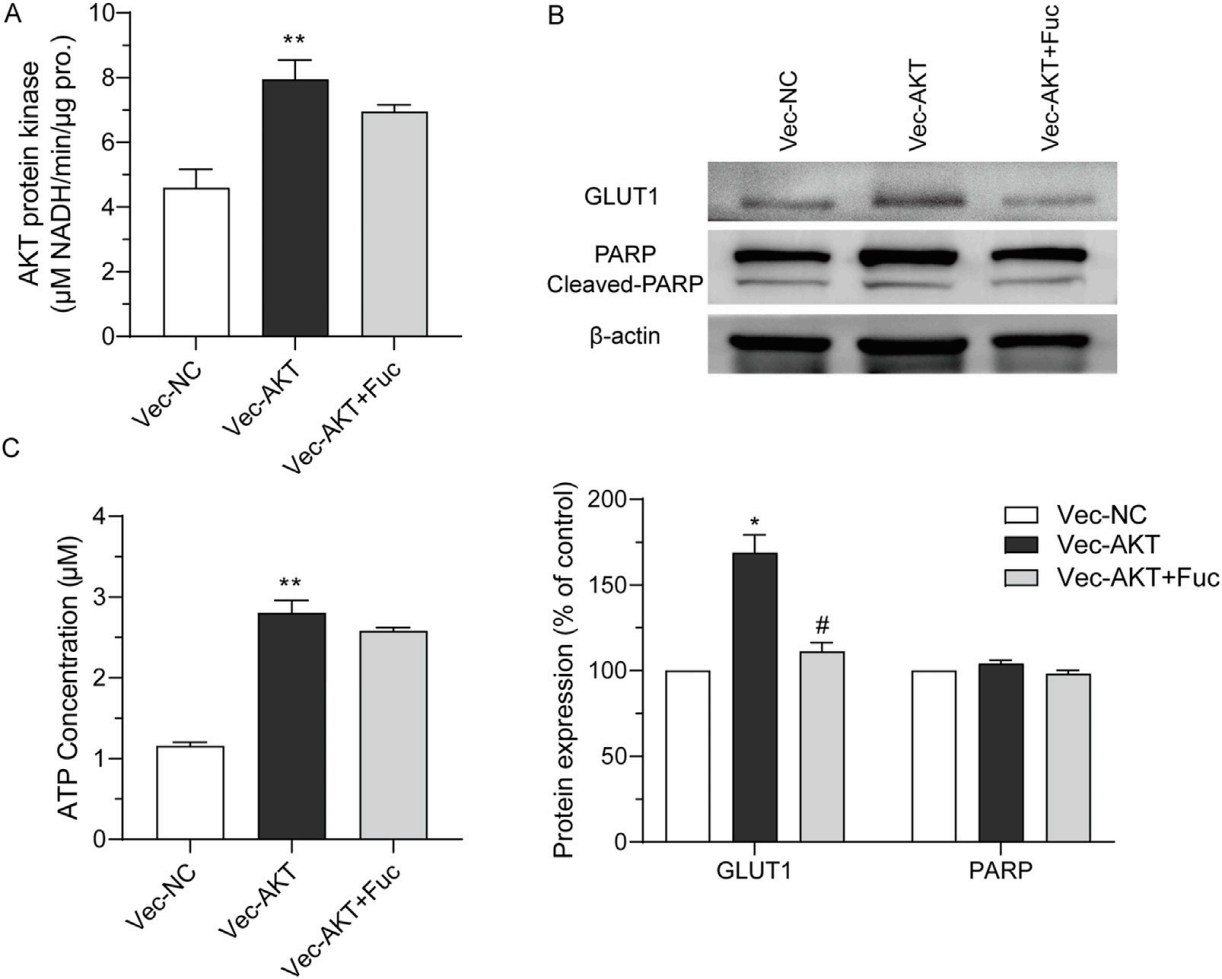
Fucoxanthin targeted AKT to inhibit ATP production and PARP activation. **(A)** AKT kinase activity in response to AKT overexpression. **(B)** The expression of related downstream proteins after AKT overexpression. **(C)** Effect of AKT overexpression on energy generation. Data are presented as the mean ± SD of three experiments. ^*^ *P* < 0.05, ^**^ *P* < 0.01 compared to Vec-NC; ^#^ *P* < 0.05 compared to Vec-AKT (n = 3).

**FIGURE 7:**
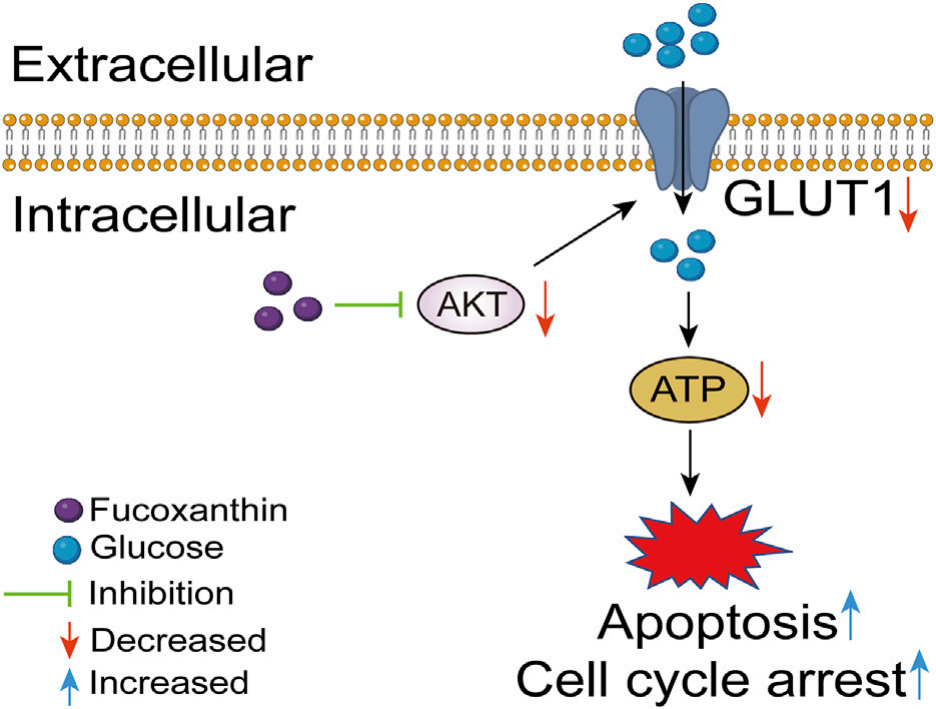
Fucoxanthin targets AKT to induce apoptosis and cell cycle arrest in MOLM13 cells.

A cell model with AKT overexpression was built to further examine the effects of fucoxanthin. The overexpression of the AKT protein increased the AKT kinase activity (*P* < 0.01, Figure 6A), upregulated GLUT1 expression (*P* < 0.05, Figures 6B,C), and increased the intracellular ATP (*P* < 0.01, Figure 6C). The AKT overexpression did not have any obvious effect on the cleavage of the PARP protein (Figure 6B). When the cell model was treated with 25 μM fucoxanthin for 24 h, the AKT kinase activity dropped by 12.6% (*P* > 0.05), the GLUT1 expression decreased by 34.1% (*P* < 0.05), although the ATP level changed very little. The activation of PARP cleavage induced by fucoxanthin was hindered after AKT overexpression (Figure 6B). The results suggested that fucoxanthin targeted the AKT protein to inhibit ATP production and activate PARP cleavage, thereby promoting cell cycle arrest and apoptosis (Figure 7).

## 4 Discussion

Fucoxanthin demonstrates a superior safety profile compared to conventional FLT3 inhibitors: Preclinical studies show no mortality or organ damage at single doses ≤2,000 mg/kg or repeated doses ≤1,000 mg/kg/day for 30 days in mice (Beppu et al., 2009), with no observed adverse effect level (NOAEL) > 200 mg/kg/day (Lau and Kwan, 2022; Peng et al., 2011). It significantly outperforms midostaurin (rodent LD_50_ 300–500 mg/kg) and gilteritinib (grade 3–4 cytopenias in 52% patients) (Perl, 2024). Clinically, 3 mg/day fucoxanthin elicits no severe adverse events in obesity trials (Hitoe and Shimoda, 2017), whereas 34% of the AML patients must discontinue midostaurin due to febrile neutropenia (Daver et al., 2021). This safety advantage aligns with the metabolism-targeting (vs. DNA-damaging) mechanism, suggesting a potentially safer option for FLT3-ITD AML. Therefore, we investigated the anti-leukemic mechanisms of fucoxanthin in this context.

Fucoxanthin exerts potent anti-cancer effects through context-dependent modulation of cell cycle arrest and apoptosis pathways. In this study, fucoxanthin induced G0/G1 phase arrest in MOLM13 cells by suppressing CDK4 expression. The findings align with observations in bladder cancer and melanoma, where cyclin D1-CDK4/6 downregulation mediates G1 arrest (Kim et al., 2013; Tafuku et al., 2012). However, in colon cancer cells, G1 arrest occurs via p21 upregulation and pRB phosphorylation without altering cyclin D/CDK4 levels, while prostate cancer models implicate GADD45A/JNK signaling (Ahmed et al., 2022). Notably, gastric adenocarcinoma uniquely exhibits G2/M arrest via cyclin B1/survivin suppression (Yu et al., 2011), highlighting the pleiotropic mechanisms of fucoxanthin.

Apoptosis induction by fucoxanthin involves multi-pathway convergence. In MOLM13 cells, we observed Bcl-2 downregulation, which is consistent with PARP cleavage reported in HL-60 leukemia (Kim et al., 2010) and U251 glioma (Wu et al., 2019). However, Bcl-2 family modulation varies across cancers: fucoxanthin reduces Bcl-xL in melanoma (Kim et al., 2013) but spares it in PC-3 prostate cancer (Kotake-Nara et al., 2005), while HL-60 cells show no Bcl-2/Bcl-xL/Bax changes (Koch et al., 2024). That is, there must be alternative triggers like ROS overproduction or survivin inhibition (Méresse et al., 2020).

Fucoxanthin treatment leads to PARP activation and ATP reduction in MOLM13 cells, consistent with energy stress-associated cell death pathways. Excessive PARP activation depletes NAD+, which subsequently impairs ATP production by disrupting glycolysis, the citric acid cycle, and mitochondrial respiration (Zhang et al., 2019). While PARP inhibition can restore NAD+ and ATP pools, its protective effects may extend beyond energy rescue (Nakajo et al., 2024; Zou et al., 2025). In our experiments, fucoxanthin treatment significantly enhanced PARP cleavage and reduced ATP levels in MOLM13 cells. Notably, this effect was attenuated in AKT-overexpressing cells, where both PARP cleavage and ATP content were partially restored. These results demonstrate that fucoxanthin regulates PARP activity in an ATP-dependent manner, likely through its upstream inhibition of AKT signaling. While the ATP depletion-PARP hyperactivation axis primarily explains fucoxanthin-induced apoptosis, the concurrent G0/G1 arrest observed in our study suggests additional crosstalk between metabolic stress and cell cycle control. Prior studies have established that energy crisis can trigger AMPK-mediated suppression of CDK4/cyclin D1 (Li et al., 2018), and AKT inhibition directly destabilizes the mRNA of cyclin D1 (Ziegler et al., 2024). Although our current data do not experimentally link glucose uptake suppression to cell cycle arrest, this mechanistic synergy warrants future investigation.

Cancer cells’ unique reliance on glucose (the Warburg effect) makes them particularly vulnerable to metabolic disruption. Compared to normal cells, FLT3-ITD AML cells have a greater demand for glucose, relying primarily on glucose for ATP (You et al., 2019). In fact, for most cancer cells, a decrease in the glucose content can directly reduce the ATP levels (Shiratori et al., 2019). The process is mediated by glucose transporters (especially GLUT1) (Heydarzadeh et al., 2020), and the increased expression of GLUT1 has been linked to lower survival rates for various types of cancers, including gastric cancer, thyroid cancer, and breast cancer (Weng et al., 2022; Xu et al., 2020). The inhibition of GLUT1 is proven to suppress tumor proliferation. For example, Yao et al. demonstrated that inhibiting GLUT1 effectively induces autophapy in hepatocellular carcinoma (Yao et al., 2023). Weng et al. used a GLUT1 inhibitor in combination with cisplatin to accomplish a synergistic effect on inhibiting the growth of breast cancer cells (Weng et al., 2022). In our study, fucoxanthin treatment significantly impaired glucose transport and uptake in MOLM13 cells, directly accounting for the observed ATP crisis. To decipher the upstream mechanism, we demonstrated that fucoxanthin directly inhibits AKT kinase activity in MOLM13 cells. This inhibition may disrupt the FLT3-AKT signaling axis, a well-characterized pathway in AML pathogenesis. The AKT pathway is an upstream signaling pathway that facilitates the translocation of GLUT to the cell surface to promote glucose uptake in various types of tumor cells (Patsoukis et al., 2017). During this translocation, AS160 acts as a substrate for the AKT protein to enable the fusion of GLUT1 vesicles with the plasma membrane, thus promoting GLUT1-mediated glucose metabolism (Heydarzadeh et al., 2020; Ni et al., 2022). We found that fucoxanthin-induced AKT inhibition led to the downregulation of GLUT1. Indeed, it has been reported that AKT regulates GLUT1 trafficking in cancer cells. For example, Ni et al. demonstrated that PI3K/AKT signaling can induce abnormal cell cycle changes and cell proliferation in the glomeruli by enhancing GLUT1-mediated glucose transport (Ni et al., 2022), and Xu et al. showed that inhibiting the PI3K/AKT pathway significantly impedes tumor growth and downregulates GLUT1 expression (Xu et al., 2020).

## 5 Conclusion

This study reveals a novel anti-leukemic mechanism of fucoxanthin in FLT3-ITD AML cells: dual action as an AKT inhibitor and metabolic modulator. Specifically, fucoxanthin (25 μM) reduced cell viability by 63.6%, downregulated GLUT1 by 36.1% (Western blot), and decreased glucose uptake by 75.35%. These findings highlight fucoxanthin’s potential to simultaneously target proliferative and metabolic pathways in FLT3-ITD AML, although the studied single-cell-line model (MOLM13) cannot fully represent FLT3-mutated AML heterogeneity and validation in primary samples is needed. Despite these limitations, the documented safety profile and the multimodal activity of fucoxanthin warrant further preclinical development.

## Data Availability

The original contributions presented in the study are included in the article, further inquiries can be directed to the corresponding authors.
